# Zebrafish Ace2N-mNeon expression toolkit for *in vivo* voltage imaging of neuronal synchrony and cardiac maturation

**DOI:** 10.1117/1.NPh.13.S2.S23205

**Published:** 2026-05-09

**Authors:** Zhenzhen Wu, Rui Oliveira Silva, Ruya Houssein, Fabiola Marques Trujillo, Jordan Gotti, Srividya Ganapathy, Zhenyu Gao, Daan Brinks

**Affiliations:** a Delft University of Technology, Department of Imaging Physics, Delft, The Netherlands; b Erasmus University Medical Center, Department of Neuroscience, Rotterdam, The Netherlands; c Erasmus University Medical Center, Department of Molecular Genetics, Rotterdam, The Netherlands

**Keywords:** bioelectricity, *in vivo* voltage imaging, genetically encoded voltage indicator (GEVIs), zebrafish, embryogenesis

## Abstract

**Significance:**

Bioelectricity is a fundamental biophysical property of cells that shapes embryogenesis through neuronal signaling and tissue patterning. Yet techniques for cell-resolved, millisecond-scale voltage imaging in early embryos remain limited.

**Aim:**

To address this need, we developed a zebrafish expression toolkit for the genetically encoded voltage indicator (GEVI) Ace2N-mNeon, which provides membrane-localized fluorescence from 4 hours post-fertilization (hpf) and supports both broad and promoter-driven, cell-type-specific expression.

**Approach:**

This toolkit is built as a small Tol2-based promoter panel, placing Ace2N-mNeon under commonly used zebrafish promoters, and can be delivered by standard Tol2 microinjection workflows. This toolkit spans multiple embryonic tissues; here, we focus on neurons and cardiomyocytes as examples.

**Results:**

Using high-speed *in vivo* voltage imaging, we recorded neuronal activity from multiple neuronal types at 1 day post-fertilization (dpf) and observed synchronous activity among motor neurons during primary neurogenesis that appeared reduced or absent during secondary neurogenesis. In parallel, from 1 to 4 dpf, atrial and ventricular voltage signals were detectable before overt chamber delineation; chamber-specific action potential propagation sharpened and accelerated with development, indicating cross-tissue generalizability.

**Conclusions:**

Collectively, these findings establish the Ace2N-mNeon expression toolkit as a practical resource for *in vivo* voltage imaging during early embryonic development, providing a foundation for linking membrane-voltage dynamics to emergent circuit-level properties.

## 1 Introduction

Bioelectricity, defined as the voltage changes across and ionic currents through cellular membranes, is a fundamental biophysical mechanism that regulates a wide range of developmental processes, including cellular differentiation, pattern formation, and morphogenesis.^1^^–^^4^ These electrical signals span multiple timescales, from millisecond-scale action potentials (APs) in excitable cells (neurons and myocytes),^5^ to slower membrane voltage ($V_{m}$) dynamics that evolve over minutes to days across diverse tissues.^1^^,^^2^ Understanding these dynamic bioelectric signals is important for elucidating their roles during embryogenesis.^1^^–^^4^^,^^6^

Neuronal APs, also known as spikes, encode and transmit information essential for processes such as sensory perception, motor control, and cognition,^5^ whereas cardiac APs coordinate contraction and reflect functional maturation of the heart.^7^ To advance our understanding of these bioelectric signals during embryogenesis, it is crucial to monitor $V_{m}$ dynamics *in vivo* across multiple cell types at early stages. However, achieving cellular resolution with millisecond timing in developing embryos remains difficult: electrophysiology is invasive and has limited capacity for simultaneous multicell recordings.^8^^,^^9^ Recent advances in genetically encoded voltage indicators (GEVIs) enable noninvasive, real-time visualization of $V_{m}$ dynamics through fluorescence changes,^10^^–^^13^ making *in vivo* monitoring of early developmental activity feasible.^14^^,^^15^ Zebrafish, with optical transparency and rapid development, are particularly well suited for high-speed *in vivo* voltage imaging during early embryogenesis.^16^^,^^17^ Nevertheless, despite several studies extending these approaches to early embryonic stages, systematic *in vivo* measurements at the earliest stages remain limited.^14^^,^^18^^,^^19^

Ace2N-mNeon provides a practical balance of sensitivity, kinetics, and brightness for high-speed *in vivo* imaging.^20^ We therefore developed a zebrafish Ace2N-mNeon expression toolkit that yields membrane-localized fluorescence from the 512-cell stage and supports both broad and promoter-driven, cell-type-specific expression. To generate this toolkit, we cloned Ace2N-mNeon into a Tol2 backbone and placed it under a small set of commonly used zebrafish promoters. Constructs were delivered by Tol2-mediated transient transgenesis following microinjection, and expression was assessed across multiple tissues and developmental stages. We document this multitissue coverage to guide future applications, while functional validation focuses on neurons and cardiomyocytes. Using high-speed *in vivo* voltage imaging, we examined activity across multiple neuronal classes at 25 hours post-fertilization (hpf), with a targeted analysis of spinal cord motor neuronal synchronous activity during the transition from primary to secondary neurogenesis. In parallel, we evaluated atrial and ventricular optical APs from 25 to 102 hpf, detecting signals prior to overt chamber delineation and observing developmental sharpening of waveforms and increased rates.

Collectively, these findings establish the Ace2N-mNeon expression toolkit as a practical tool for cell-resolved, millisecond-scale *in vivo* voltage imaging during early embryogenesis and demonstrate cross-tissue generalizability. This resource provides a foundation for studies linking membrane-voltage dynamics to emergent circuit-level properties at single-cell resolution.

## 2 Results

### 2.1 Ace2N-mNeon Predominantly Localizes to the Plasma Membrane Across Diverse Cell Types During Zebrafish (*Danio rerio*) Development

**Fig. 1:**
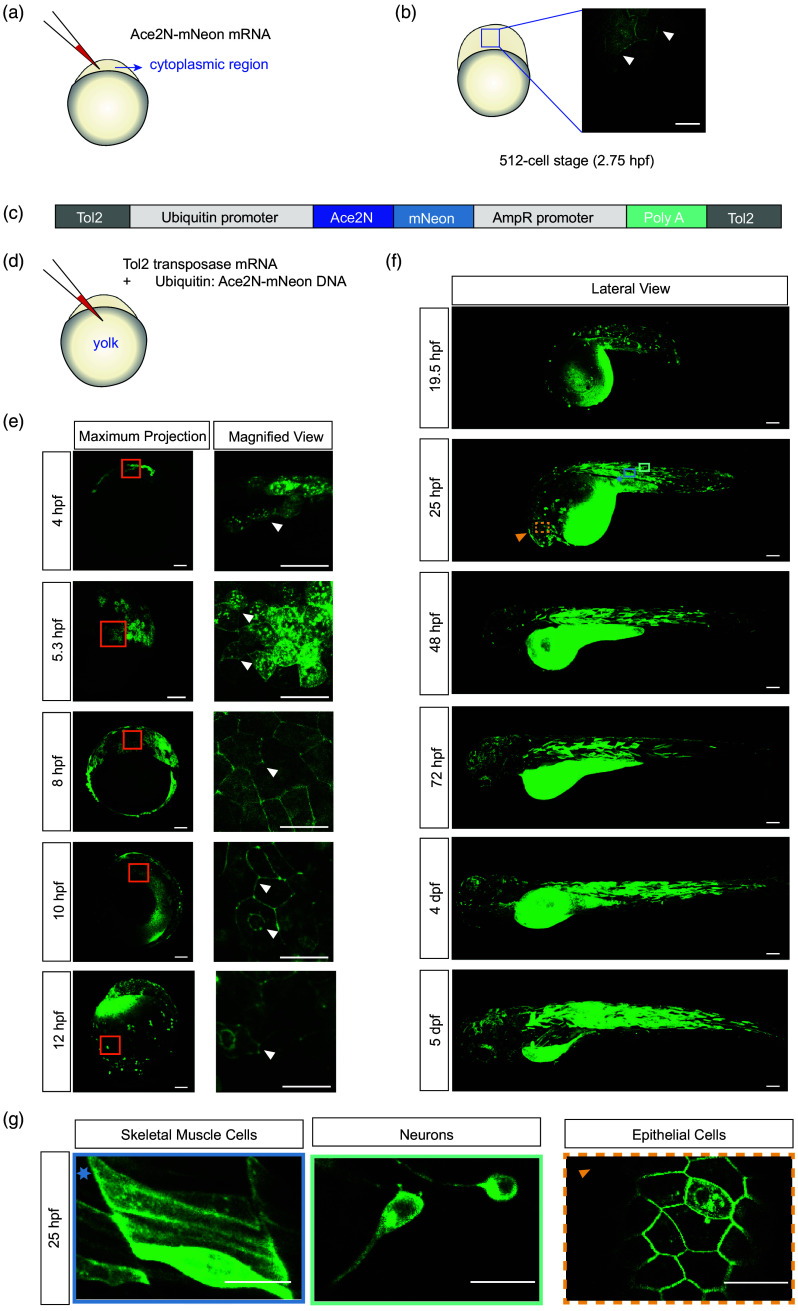
Expression of Ace2N-mNeon in various cell types throughout development. (a) Schematic of microinjection of Ace2N-mNeon mRNA into the cytoplasm of a one-cell stage embryo. (b) Single z-plane confocal image of Ace2N-mNeon mRNA expression at the 512-cell stage. Membrane localization can be observed at 2.75 hpf (white arrows). Scale bar=20  μm. (c) Diagram of Tol2 constructs driving Ace2N-mNeon from the ubi promoter. (d) Schematic of co-injection of ubi: Ace2N-mNeon Tol2 plasmid and Tol2 transposase mRNA into the yolk at the one-cell stage. Panels (e)–(f) show maximum-intensity projections under the ubi promoter from the sphere stage (4 hpf) to 5 dpf. Ace2N-mNeon is widely expressed with predominant membrane localization across various cell types. (e) Early expression from 4 to 12 hpf. Left: Maximum-intensity projection, scale bar=100  μm. Right: Magnified view showing membrane labeling, scale bar=50  μm. (f) Lateral views of Ace2N-mNeon expression in multiple cell types from 1 to 5 dpf. Scale bar=100  μm. (g) Single z-plane confocal image of Ace2N-mNeon expression at 25 hpf. Distinct cell types can be identified based on morphology, including skeletal muscle cells, neurons, and epithelial cells. Scale bar=20  μm. hpf: hours post-fertilization; dpf: days post-fertilization; ubi: ubiquitin.

To study bioelectricity during early development, we established strategies to express Ace2N-mNeon across developmental stages. To capture the earliest expression, we injected Ace2N-mNeon mRNA into the cytoplasm of one-cell embryos [Fig. 1(a)]. Membrane-localized fluorescence was detectable as early as 2.75 hpf, coincident with the onset of the blastula stage [Fig. 1(b)]. Unless stated otherwise, all data shown are from transiently injected embryos.

To follow expression throughout development, we used the Tol2 transposon system (see Sec. 4).^21^ First, we examined broad Ace2N-mNeon expression driven by the ubiquitin (ubi) promoter, which supports multitissue expression throughout zebrafish development, with variability typical of transient expression.^22^ We constructed a *ubi: Ace2N-mNeon* Tol2 plasmid [Fig. 1(c)] and co-injected it with Tol2 transposase mRNA into the yolk of one-cell embryos [Fig. 1(d); Figs. S1 and S2 in the Supplementary Material].

Ace2N-mNeon was broadly expressed across cell types throughout development [Figs. 1(e) and 1(f)].^23^^,^^24^ Ace2N-mNeon-positive embryos could be detected starting from the sphere stage (4 hpf) using confocal microscopy. During gastrulation (50% epiboly, 5.3 hpf, to embryonic shield, 10 hpf), expression persisted in newly differentiating cells with strong membrane fluorescence [Fig. 1(e), 4 to 8 hpf]. From 10 hpf through the 21-somite stage (19.5 hpf), as segmentation began and somites formed [Fig. 1(e), 10 to 12 hpf], the embryo developed a more defined body shape [Fig. 1(f), 19.5 hpf]. By the prim-6 stage (25 hpf), the embryo exhibited a recognizable body plan, including head, tail, and clear segmentation [Fig. 1(f)]. At this stage, multiple cell types were identifiable by morphology, including skeletal muscle, neural, and epithelial cells, with broad Ace2N-mNeon expression and strong localization to the cell membrane [Fig. 1(g)]. This expression can be observed throughout development, at least 5 days post-fertilization (dpf). More detailed expression images from 1 to 5 dpf can be found in Fig. S1 in the Supplementary Material. In transient Tol2 embryos, broad promoters still yield mosaic expression due to stochastic integration; moreover, membrane-localized signals can appear highly nonuniform across tissues because intensity depends on cell type, membrane area, and developmental stage.

Together, these findings demonstrate stable, widespread expression with predominant membrane localization of Ace2N-mNeon from gastrulation onward, persisting across diverse cell types through 5 dpf.

### 2.2 Cell-Specific Promoters Drive Differential Ace2N-mNeon Expressions During Zebrafish (*Danio rerio*) Development

In our initial experiments, we established ubiquitous Ace2N-mNeon expression across zebrafish development, providing membrane-targeted labeling across multiple cell types and stages. We then built promoter-driven, cell-type-specific constructs, facilitating targeted investigation of bioelectrical activity in defined cell types (Table 1). Given the crucial roles of neurons and myocytes during development, our research primarily focused on these cell types. Neurons are essential for propagating bioelectric signals that orchestrate tissue and organogenesis,^2^ whereas myocytes are integral to motility, structural support, and cardiac functionality.^25^

**Table 1:** Cell-specific promoters used to drive Ace2N-mNeon expression in zebrafish.

Promoter (gene)	Target cell types
*503-bp unc-45b*	Skeletal and cardiac muscle cells
*acta2* (α-Smooth muscle actin)	Smooth muscle cells and pericytes
*cmlc2* (cardiac myosin light chain 2)	Cardiomyocytes
e*lavl3* (ELAV-like protein 3)	Neurons
*neuroD* (Neurogenic differentiation 1)	Neurons

**Fig. 2:**
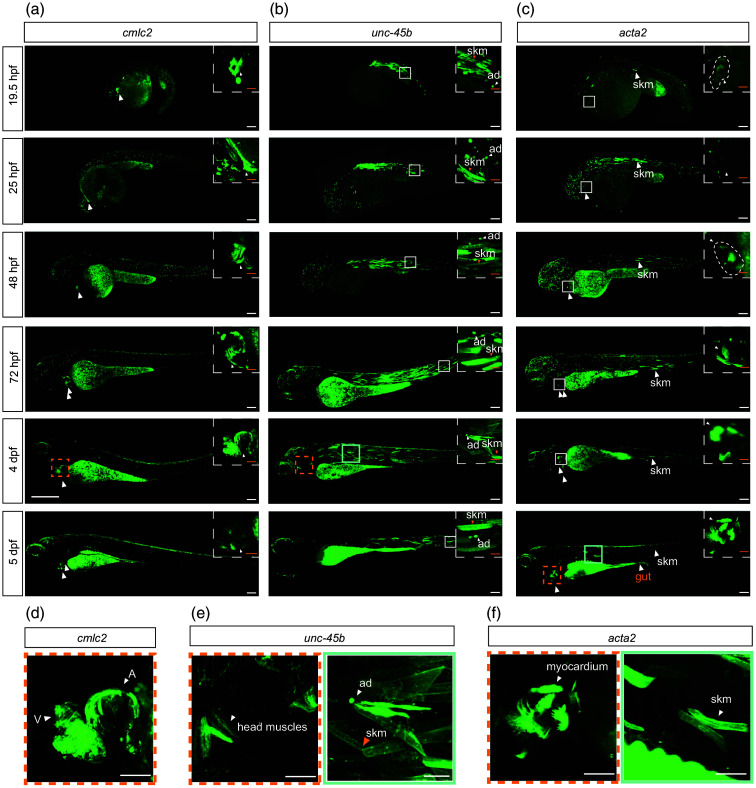
Ace2N-mNeon expression in muscle cell types across developmental stages. Panels (a)–(c) show whole-embryo maximum-intensity projections of Ace2N-mNeon driven by the indicated promoters from 19.5 hpf to 5 dpf. Scale bar=100  μm. Boxed regions are shown at higher magnification (scale bar=25  μm). (a) cmlc2: cardiomyocytes labeled from the 21-somite stage (19.5 hpf), with progressively stronger signal over time. (b) unc-45b: stronger labeling in skm (orange arrows) and ad (white arrows); head musculature is visible by 4 dpf (orange dashed box). (c) acta2: sparse labeling at 19.5 hpf; from 48 hpf, labeling appears in the myocardium, and by 5 dpf, a robust signal is present in the myocardium (orange dashed box) and visceral smooth muscle of the gut (orange arrows). Panels (d)–(f) show higher-magnification single z-plane images at later stages. Scale bar=50  μm. (d) cmlc2 at 4 dpf: cardiomyocytes with clear labeling in the A and V. (e) unc-45b at 4 dpf: head muscles (orange dashed box), skeletal muscle (green box; orange arrows), and ad (green box; white arrows). (f) acta2 at 5 dpf: labeling in the myocardium (orange dashed box) and visceral smooth muscle (green box). Panels use different intensity scaling; yolk intensity should not be compared across panels. hpf: hours post-fertilization; dpf: days post-fertilization; skm: skeletal muscle cells; ad: adaxial cells; A: atrium; V: ventricle.

**Fig. 3:**
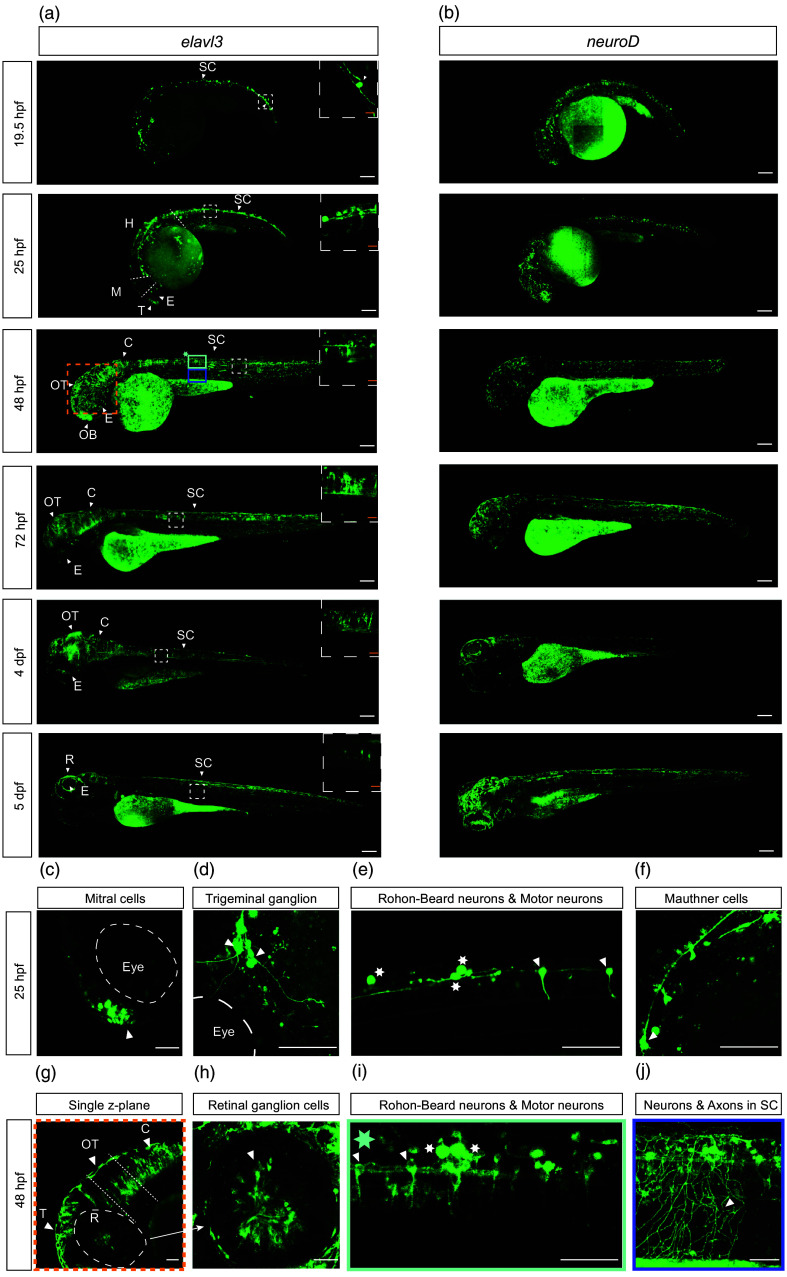
Ace2N-mNeon expression in neurons across developmental stages. Panels (a)–(b) show whole-embryo maximum-intensity projections of Ace2N-mNeon driven by the indicated neuronal promoters from 19.5 hpf to 5 dpf. Scale bar=100  μm. Boxed regions are shown at higher magnification as single z-plane confocal images from the indicated brain region. Scale bar=20  μm. (a) elavl3: prominent labeling in SC by 19.5 hpf, extending to T and H by 25 hpf, and increased in OB, R, OT, and C from 2 to 4 dpf. (b) neuroD: labeling is sparser compared with elavl3. Panels (c)–(f) show single z-plane images at 25 hpf showing identifiable neuronal populations by location and morphology. Scale bar=50  μm. (c) Mitral cell in the T (white arrows). (d) Trigeminal ganglion neurons (white arrows). (e) RB mechanosensory neurons (white stars) and motor neurons (white arrows). (f) Mauthner cells (white arrows). (g–j) Single z-plane images at 48 hpf. Scale bar=50  μm. (g) Labeling in the R, OT, and C (region indicated by the orange dashed box in a). (h) RGCs with centrally projecting axons. (i) Neurons and axons in the spinal (regions indicated by the asterisk-labeled green box in a). (j) Secondary motor neuron axons projecting to ventral trunk muscles. Panels use different intensity scaling; yolk intensity should not be compared across panels. hpf: hours post-fertilization; dpf: days post-fertilization; SC: spinal cord; T: telencephalon; H: hindbrain; OB: olfactory bulb; R: retina; OT: optic tectum; C: cerebellum; RB: Rohon-Beard; RGCs: retinal ganglion cells.

Zebrafish embryogenesis is marked by rapid development, with somite formation beginning around 16 hpf and the basic body plan and early organ systems forming by 19.5 hpf.^26^ To investigate bioelectricity changes during this window, we assessed promoter-driven expressions from the 21-somite stage (19.5 hpf), a key point for early organogenesis.^26^ As expected, constructs yielded cell-specific expression; expression levels were mosaic and varied across embryos and cell types, consistent with Tol2-mediated transient transgenesis and promoter-dependent strength (Figs. 2 and 3). In embryos with ubiquitous expression, overall fluorescence is higher, and the yolk can appear very bright. In neuronal and cardiac preparations, stage-dependent autofluorescence (consistent with uninjected controls in the Fig. S2 in the Supplementary Material) reduces contrast, so later-stage panels are displayed with higher contrast.

#### 2.2.1 Ace2N-mNeon expression in muscle cells

Zebrafish musculature comprises smooth, skeletal, and cardiac muscle cells, each with distinct gene expression profiles and localizations.^25^ The promoters used in this study allow the differentiation and independent analysis of these cell types.

Expression in cardiac muscle cells was driven by the promoter for cardiac myosin light chain 2 (*cmlc2*), which is a key cardiac contractile protein. The zebrafish *cmlc2* promoter drives specific expression in cardiomyocytes.^27^ The zebrafish *cmlc2* promoter allows specific expression in cardiomyocytes.^28^ The expression begins at 19.5 hpf and continues throughout development [Fig. 2(a)]. By 4 dpf, distinct atrium (A) and ventricle (V) structures are observable [Fig. 2(d)], allowing separate recording of electrical dynamics.

To investigate skeletal muscle cells (skm), we used the promoter for *unc-45b*, a myosin chaperone that is important for the correct assembly of the contractile apparatus in developing muscles.^29^ In zebrafish, it is specifically expressed in striated muscle, including both cardiac and skeletal muscles, where it can help myosin fold during myofiber formation.^29^^,^^30^ At 19.5 hpf, the *unc-45b* promoter labels skm and adaxial cells (ad, progenitors of slow-twitch skm) [Fig. 2(b), 19.5 hpf]. These cells can be identified by their morphology [Figs. 2(b) and 2(e)]. By 3 dpf, fluorescence was evident in head muscles [Fig. 2(e)].

Smooth muscle cells (SMCs) were labeled using the promoter for α-smooth muscle actin (*acta2) expression. Acta2* is one of the earliest markers for mural cell development in vertebrates, expressed in SMCs and pericytes.^31^ In zebrafish, *acta2: EGFP* expression begins slightly later in development, initially in the myocardium, followed by visceral smooth muscle and skeletal muscle, and eventually in vascular smooth muscle.^31^ At 19.5 hpf, *acta2* expression is faintly detectable in the myocardium and in a few skm [Fig. 2(c), 19.5 hpf]. More expressions in skm could be observed from 25 hpf onwards [Fig. 2(c), 25 hpf]. By 48 hpf, the *acta2* signal was observed in the myocardium, consistent with early *acta2* reporter activity [Fig. 2(c), 48 hpf]. By 5 dpf, strong expression was observed in the myocardium and visceral smooth muscle of the gut [Figs. 2(c) and 2(f), 5 dpf].

#### 2.2.2 Ace2N-mNeon expression in neurons

During early somite formation, *elavl3*, an mRNA-binding protein broadly expressed in newly differentiated neurons,^32^ is detectable in the neural tube and forebrain of zebrafish embryos [Fig. 3(a)]. Under the *elavl3* promoter, Ace2N-mNeon labeling is prominent by 1 dpf in the spinal cord (SC) and telencephalon (T) [Fig. 3(a); 19.5 hpf, 25 hpf]. Additional brain region-resolved views across development are provided in Fig. S3 in the Supplementary Material. At 25 hpf, distinct neuronal populations can be identified by location and morphology, including mitral cells,^33^ trigeminal ganglion neurons,^34^ Mauthner cells,^35^ Rohon-Beard mechanosensory (RB) neurons,^36^ and motor neurons^37^ [Figs. 3(c)–3(f)].

By 2 dpf, Ace2N-mNeon labeling expands across the central nervous system (CNS). In addition, regions visible at 1 dpf show signals in the retina (R), optic tectum (OT), and cerebellum (C) [Figs. 3(a) and 3(g)]. At this stage, retinal ganglion cells (RGCs) are identifiable with centrally projecting axons [Fig. 3(h)], and secondary motor neurons projecting to the ventral trunk muscles also become distinguishable [Figs. 3(i) and 3(j)]. From 2 to 4 dpf, labeling density increases in the OT, C, and SC; individual neurons become harder to resolve in maximum-intensity projections but remain distinguishable in single z-planes from 3D scans [Fig. 3(a), white dashed box]. Ace2N-mNeon expression persists in the CNS at least through 5 dpf.

We compared *elavl3*- and *neuroD-driven expression* [Figs. 3(a) and 3(b)].^38^ Labeling under *elavl3 was* denser than under the *neuroD* [Figs. 3(a) and 3(b)], consistent with their endogenous profiles: *elavl3* is broadly expressed in newly differentiated neurons,^39^ whereas *neuroD* labels subsets and is not universal across neuronal classes.^40^ These observations indicate that Ace2N-mNeon can be targeted to defined neuronal populations with membrane localization throughout early development.

### 2.3 Ace2N-mNeon Voltage Imaging Reveals Neurogenesis-Specific Electrical Activity

To validate the library for studying neural development *in vivo*, we performed high-speed imaging of identified neuronal types and analyzed activity traces using a standard motion correction and spike extraction workflow. We co-injected the *elavl3:Ace2N-mNeon* construct with Tol2 transposase mRNA into one-cell-stage embryos and selected labeled embryos for high-speed imaging in defined brain regions from 24 to 28 hpf.

**Fig 4:**
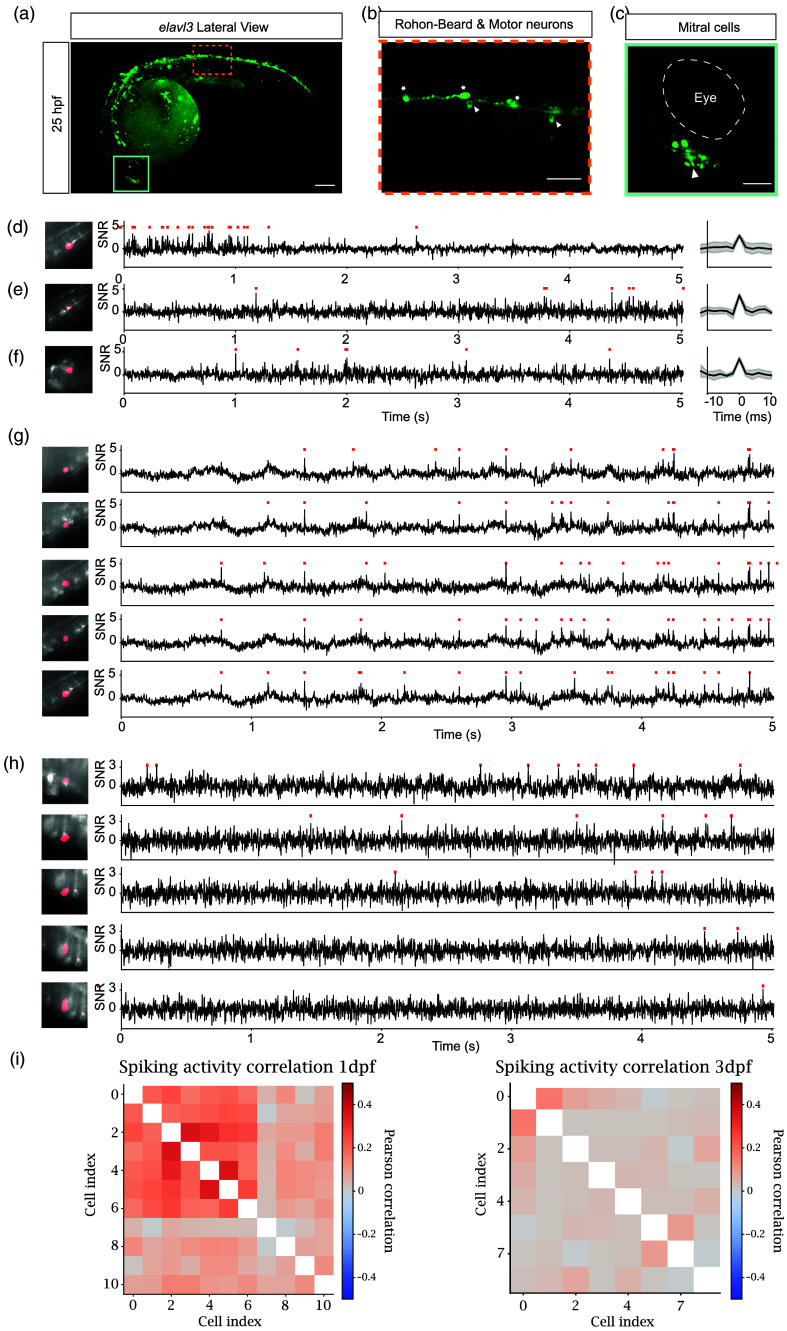
Ace2N-mNeon voltage across neuronal classes during early development. (a) Whole-embryo maximum-intensity projections of Ace2N-mNeon driven by elavl3 at 25 hpf. Scale bar=100  μm. Orange and green boxes indicate regions shown at higher magnification, as shown in (b) and (c). (b) Single z-plane in the spinal cord showing motor neurons (white arrows) and RB neurons (white stars). Scale bar=50  μm. (c) Single z-plane in the olfactory bulb showing mitral cells (white arrow). Scale bar=50  μm. Panels (d)–(f) show voltage recordings at 25 hpf from the three neuronal classes. Left: mean image of the recording with spatial footprint. Center: example fluorescence traces. Right: average spike waveform with standard deviation from detected events. (d) Motor neuron. (e) RB neuron. (f) mitral cell. Panels (g)–(h) show voltage recordings from multiple motor neurons in the same field of view at ∼25  hpf (g) and 3 dpf (h). Left: mean with spatial footprints, ROI was manually annotated in red. For all traces, red ticks indicate detected spikes. (i) Correlation matrices of spiking activity recorded simultaneously at 1dpf (left) and 3dpf (right). RB: Rohon–Beard; ROI: region of interest; hpf: hours post-fertilization; dpf: days postfertilization.

For voltage imaging, we targeted three identifiable neuronal classes based on morphology and location: RB mechanosensory neurons, spinal motor neurons, and mitral cells in the olfactory bulb [Figs. 4(a)–4(c)]. Along the tail, motor neurons were recognized by axons projecting to the ventral myotome,^37^ and RB neurons by laterally extended axons [Fig. 4(b)]; in the rostral bulb near the eyes, mitral cells were located adjacent to the medial olfactory tract [Fig. 4(c)].^33^ Voltage transients were detectable at 25 hpf in all three classes [Figs. 4(d)–4(f)].

During zebrafish neurogenesis, a primary scaffold precedes secondary neurogenesis, which involves axonal and dendritic elaboration and the emergence of early synapses.^41^^–^^43^ To examine activity across this transition, we imaged sparsely labeled spinal motor neurons at 1 and 3 dpf, identifying individual cells in the recording plane. Across embryos, neighboring motor neurons showed more frequent synchronized activity at ∼25  hpf that appeared reduced or absent at 3 dpf [Figs. 4(g)–4(h)], indicating that distinct activity patterns across primary versus secondary neurogenesis.^42^^,^^43^ With continuous expression of the indicator, regions became denser and signal-to-background ratio (SBR) decreased [Figs. S4(a)–S4(d) in the Supplementary Material], leading also to a decrease in spike detection signal-to-noise ratio (SNR) [Fig. S4(e) in the Supplementary Material]. Given the dense labeling and resulting noisy traces, we studied false positive spikes by performing spike detection in the inverted trace, using the same SNR-based detection threshold as in the native trace. As expected, false positive spike detection increases with decreasing SNR [Fig. S4(f) in the Supplementary Material]; in densely expressing areas, only high SNR traces should therefore be used for analysis. These observations indicate that Ace2N-mNeon supports *in vivo* voltage recordings across neuronal classes during early development and can reveal qualitative changes in motor network synchronization during neurogenesis.

### 2.4 Detection of Development-Dependent Voltage Dynamics in the zebrafish Heart

**Fig. 5:**
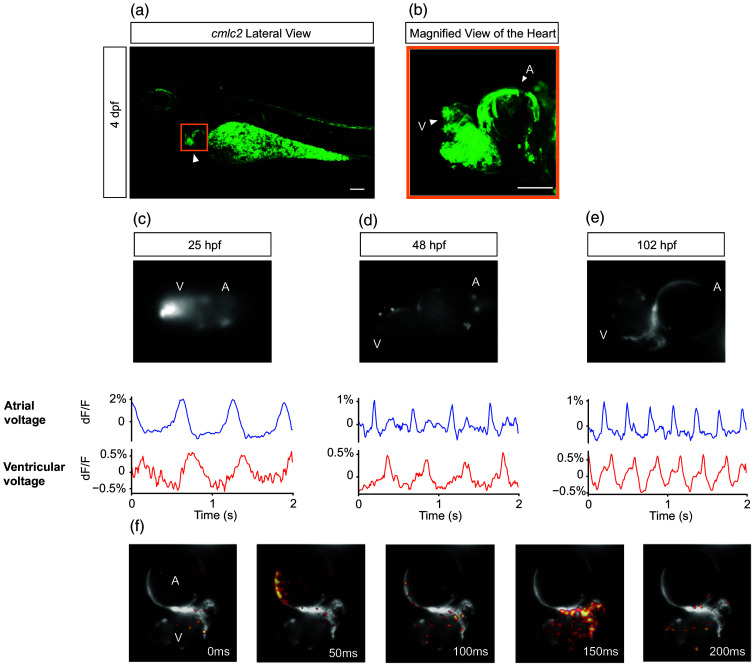
Atrial and ventricular voltage dynamics in the embryonic zebrafish heart. (a) Maximum-intensity projections of Ace2N-mNeon driven by cmlc2 at 4 dpf. Scale bar=100  μm. (b) Higher-magnification view of the boxed region in (a), showing Ace2N-mNeon membrane-localized and clear delineation of the A and V at 4 dpf. Scale bar=50  μm. Panels (c)–(e) show developmental series of A and V optical voltage recordings at 25, 48, and 102 hpf. Top: fluorescence images of the recorded hearts (25 hpf tubular heart without overt chamber separation; 48 hpf clearly delineated chambers). Voltage recordings and ROI placement were performed on a single z-plane. Bottom: optical voltage traces (ΔF/F) from A and V at (c) 25 hpf, (d) 48 hpf, and (e) 102 hpf. Illumination was minimized to limit blue-light inactivation of pAB; 25 hpf traces exhibit lower signal-to-noise due to weaker expression. (f) Voltage-time footprint (temporal-maximum ΔF/F) over a 200-ms window at 102 hpf; color encodes peak ΔF/F relative to baseline. Together with the atrial and ventricular traces, the spatial pattern is consistent with A–V propagation. Low-frequency wiggles likely reflect residual motion and are minimized by ROI selection and exclusion of contracting segments. hpf: hours post-fertilization; dpf: days post-fertilization; A: atrium; V: ventricle; pAB: para-aminoblebbistatin; ROI: region of interest. (a) Video 1, MOV, 2.15 MB [URL: https://doi.org/10.1117/1.NPh.13.S2.S23205.s1]. (c) Video 2, MOV, 21.4 MB [URL: https://doi.org/10.1117/1.NPh.13.S2.S23205.s2]. (d) Video 3, MOV, 3.90 MB [URL: https://doi.org/10.1117/1.NPh.13.S2.S23205.s3]. (e) Video 4, MOV, 3.81 MB [URL: https://doi.org/10.1117/1.NPh.13.S2.S23205.s4].

Using the same expression resource applied to neurons, *cmlc2: Ace2N-mNeon* showed strong membrane labeling in cardiomyocytes from the 21-somite stage [Fig. 2(a)], enabling *in vivo* optical readout of cardiac activity. At 4 dpf, lateral views reveal persistent and pronounced membrane localization, with a visibly stronger signal than at earlier stages [Figs. 5(a) and 5(b)].

To study cardiac AP dynamics during early development, we imaged *cmlc2: Ace2N-mNeon*-positive embryos of the zebrafish heart at 25, 48, and 102 hpf [Figs. 5(c)–5(e)], using different embryos at each time point. To minimize motion artifacts caused by heart contractions, embryos were incubated with *para*-aminoblebbistatin (pAB, 100  μM) for 4 h before imaging,^44^ which resulted in an apparent absence of heartbeat and consequent minimal motion artifacts (Video 1). Illumination was kept low to avoid blue-light inactivation of pAB, and beating resumed promptly upon brief high-intensity blue light after each recording, confirming viability during the session (Videos 2, 3, and 4).

At 25 hpf, the heart presents as a narrow tube without an overt anatomical division [Fig. 5(c), top], yet distinctive atrial- and ventricular-type optical APs were detectable, with atrial to ventricular propagation [Fig. 5(c), bottom]. By 48 hpf, the A and V are anatomically delineated [Fig. 2(a), 48 hpf], and both compartments exhibit sharper waveforms and higher frequency [Fig. 5(d)]. This sharpening and acceleration continue through development until 102 hpf [Fig. 5(e)], while maintaining a clear A-V propagation pattern [Figs. 5(e) and 5(f)].

These *in vivo* optical recordings were consistent with prior patch-clamp measurements on explanted hearts,^45^^,^^46^ demonstrating that chamber-specific cardiac APs arise before overt morphological chamber formation. Together with the neuronal recordings, the cardiac results demonstrate the cross-tissue applicability of the Ace2N-mNeon expression toolkit for cell-resolved, early embryonic voltage imaging.

## 3 Discussion and Conclusion

GEVIs have revolutionized the study of bioelectric signals, but applications in early embryogenesis remain comparatively limited.^14^^,^^18^^,^^19^ Here, we introduce a zebrafish Ace2N-mNeon expression toolkit that delivers membrane-targeted fluorescence from the 512-cell stage and supports promoter-driven, cell-specific expression at later stages, enabling cell-resolved, millisecond-scale *in vivo* voltage imaging during early development. To our knowledge, this work presents the first demonstration of Ace2N-mNeon expression across multiple cell types in zebrafish,^47^ documenting neurons, cardiomyocytes, mural cells, and skeletal muscle cells, thereby providing a unified library for cross-tissue *in vivo* voltage imaging.

A primary application of this toolkit is tracking neuronal activity in the early developing brain. Zebrafish neurogenesis comprises a primary phase (to ∼2  dpf) that establishes a transient scaffold, followed by secondary neurogenesis with axonal/dendritic elaboration and the emergence of early synapses.^42^^,^^43^^,^^48^ During primary neurogenesis, developing neural networks often exhibit spontaneous patterned activity; synchrony has been reported to occur with phases of axon outgrowth and the emergence of early synapses.^43^^,^^48^^,^^49^ Prior approaches have provided important insights, but simultaneously, cell-resolved voltage readouts in early embryos remain challenging.^50^ In this study, we have shown that adjacent spinal cord motor neurons exhibit synchronous activity during primary neurogenesis and appear absent by 3 dpf across embryos, consistent with the transition from primary to secondary neurogenesis. These observations highlight the feasibility of *in vivo* voltage imaging to probe activity motifs across neurogenesis.

Although much of the progress in voltage imaging has centered on neural signals, Ace2N-mNeon also captured the longer-duration APs of cardiomyocytes.^51^ Here, we observed atrial and ventricular voltage activity as early as 25 hpf, even before a clearly visible anatomical division between the chambers. Moreover, as development proceeded, both atrial and ventricular APs exhibited sharper waveforms and higher frequency, which is consistent with the rise in embryonic heart rate^52^^,^^53^ and likely reflects maturation of ion-channel composition and coupling; possible contributors include increased expression of the cardiac sodium channel NaV1.5.^54^ Our optical recordings align with prior electrophysiological measurements in embryonic preparations, supporting the view that electrophysiological maturation precedes morphological partitioning.^45^^,^^46^^,^^55^ Together with the neuronal data, this supports the cross-tissue applicability of our Ace2N-mNeon toolkit.

Beyond fast spiking in excitable cells, slow $V_{m}$ changes in nonexcitable cells potentially function as endogenous cues for growth and morphogenesis, influencing both individual cell behavior and anatomical features.^2^^,^^3^^,^^6^^,^^47^ Although calibrated $V_{m}$ measurements *in vivo* remain challenging,^56^^–^^59^ the early, membrane-targeted, and broadly distributed expression achieved by our library provides a practical entry point for future studies of slow bioelectric signaling during embryogenesis as optical voltage methodologies continue to advance.

This study has several limitations. First, the neuronal analyses were mostly qualitative and based on sparsely labeled fields, because the promoters used were not population-specific. Second, signal-to-background ratio of neuronal expression declined by 3 dpf, resulting in a consequent SNR decrease, restricting longitudinal tracking primarily to motor neurons. In our transient Tol2 preparations, this decline likely reflects a combination of factors, including higher indicator expression and a higher number of cells expressing the indicator, which reduced optical contrast at later stages as tissues thickened and background increased.^60^^,^^61^ Third, Tol2-mediated transient expression produced embryo-to-embryo mosaicism, reducing comparability across individuals, and prevented assessment of cross-generation reproducibility.^21^

These limitations point to clear technical improvements. Where longer-term tracking and better comparability are needed, generating stable lines and using promoters better suited for later stages should improve consistency across clutches and reduce mosaicism.^62^ Signal quality should also improve with optimized microinjection parameters (for example, DNA concentration and injection volume), better control of expression density, and stronger background rejection (for example, improved optical sectioning).^62^^–^^64^

Looking ahead, several extensions could broaden applicability beyond these implementation limits. The promoter toolkit strategy described here is transferable, and the same panel and workflow can be applied to updated GEVIs as they become available. Volumetric light-sheet microscopy and faster detectors could expand the field of view while preserving temporal resolution.^64^ Adopting standardized synchrony metrics and shared analysis pipelines will facilitate cross-stage and cross-lab comparisons. In addition, all-optical paradigms, pairing this toolkit with optogenetic pacing or perturbations, may help establish causality in early circuit assembly and cardiac conduction.^14^ Finally, extending functional assays to additional cell types already represented in the panel will test and broaden its cross-tissue applicability.

Together, the Ace2N-mNeon expression toolkit enables *in vivo* voltage imaging across neuronal and cardiac tissues in early embryogenesis, establishes feasibility and cross-tissue applicability, and provides a practical path to chart how membrane-voltage dynamics evolve from cells to circuits in the intact embryo.

## 4 Appendix A: Materials and Methods

### 4.1 Zebrafish Husbandry

All experiments used *Casper* zebrafish (*Danio rerio*).^65^ Adults were maintained in recirculating systems at the Delft University of Technology fish facility under a 14 h light/10 h dark cycle at 28°C and pH 7.5.

### 4.2 Plasmid Construction

Ace2N-mNeon was amplified from YA1611: mNeon-Ace (a gift from Adam Cohen) and fused to various promoters in Tol2 backbones. Inserts were generated by overlap-extension PCR using Phusion high-fidelity DNA polymerase (NEB), and promoter-containing backbones were amplified with KOD Xtreme Hot Start DNA Polymerase (Merck Sigma). Fragments were assembled with Gibson Assembly Master Mix (NEB) and transformed into NEB^®^ 5-alpha competent *E. coli*. Primer sequences are listed in the Supplementary Material (Table S1 in the Supplementary Material), and all the plasmids generated in this study are listed in Table S2 in the Supplementary Material.

### 4.3 Tol2 Transposon System

The Tol2 system mediates the cut-and-paste genomic integration and supports stable transgene expression in zebrafish.^21^ Following microinjection, Tol2 transposase mRNA drives integration of the Tol2-flanked expression cassette, improving expression breadth versus episomal plasmids.^21^

### 4.4 RNA Synthesis

Ace2N-mNeon was subcloned downstream of an SP6 promoter (from *pCS2FA-CO-Tol2-TPase*, Addgene: #133032). Tol2 transposase mRNA was transcribed directly from *pCS2FA-CO-Tol2-TPase*. *In vitro* transcription was used with the mMESSAGE mMACHINE^®^ Kit (Thermo Fisher Scientific) following the manufacturer’s protocol. The synthesized RNA was purified and quantified before using subsequent microinjections.

### 4.5 Microinjection

Fertilized eggs were collected at the one-cell stage immediately after spawning. For mRNA injections, Ace2N-mNeon mRNA (100  ng/μL) with 0.1% phenol red (Sigma-Aldrich, P0290) was injected into the cytoplasm at the 1 to 4 cell stage (∼1  nL). For DNA injections, one-cell-stage embryos were co-injected into the yolk with ∼1  nL containing 50  ng/μL plasmid DNA, 100  ng/μL Tol2 transposase mRNA, and 0.1% phenol red. After injection, the embryos were incubated in egg water (Instant Ocean salts, 60  μg/mL) containing 0.0002% methylene blue. Only fertilized eggs were selected for microinjection; embryos that subsequently failed to develop normally or died were removed at 6 and 24 hours post-injection. Embryos were visually screened before imaging, and those with obvious morphological abnormalities were excluded from imaging and downstream analysis.

### 4.6 Confocal Imaging Sample Preparation

All data in this study were obtained from Tol2-mediated transient expression following microinjection; no stable transgenic lines were generated. Embryos 2.75 to 12 hpf were imaged directly with a drop of egg water. Embryos from 19.5 hpf-5 dpf were manually dechorionated (before 2 dpf) and mounted in 1.5% agarose (Sigma-Aldrich, A4018) with 0.03% tricaine (Sigma-Aldrich, A5040). Embryos at each developmental stage were separate individuals. Before and after imaging, embryos were checked for heartbeat, overall health, and gross morphology.

### 4.7 Confocal Imaging Setup

Confocal imaging used a Nikon Ti C2si inverted microscope with 485 nm excitation (illumination power ∼3.35  mW). The system was equipped with a 405/488/561/640 quad-band dichroic (Nikon, MHE46410, Tokyo, Japan); imaging used the 488-nm channel with a 525/50 emission filter (Nikon, MHE46710). Images were acquired with a 20×/0.75 NA objective (Nikon CFI Plan Fluor 20XC MI). Data acquisition was performed using NIS-Elements, and analysis was conducted with ImageJ.

All voltage recordings (neuronal and cardiac) in this study were performed on a single z-plane at the recording plane; whole-embryo maximum-intensity projections were used only for anatomical orientation in expression figures.

### 4.8 Expression Assessment

Expression was assessed by manually evaluating fluorescence in the expected target cell population for each promoter. Embryos were scored as positive when fluorescence was clearly detectable in the target cells, identified by stereotyped position and morphology, with membrane-localized signal where applicable. Yolk fluorescence and diffuse background were not used as evidence of Ace2N-mNeon expression. Autofluorescent regions present in uninjected controls were used as a reference and were excluded from scoring.

### 4.9 Voltage Imaging of Neurons

Embryos at 24 to 28 hpf and 3 dpf were paralyzed with α-Bungarotoxin (1  mg/mL, Sigma-Aldrich, 203980; 15 min) to block nicotinic receptors and suppress motion artifacts during imaging. Embryos were mounted in 1.5% low-melting agarose (Sigma-Aldrich, A4018). Single-photon voltage imaging was performed on a Nikon A1RMP microscope using a 25×/1.1 NA water-immersion objective (Nikon CFI75 Apo, 25XC W). Illumination was provided by a white LED (SOLA Light Engine, Lumencor; 20 to $50\;\mathrm{mW}/{\mathrm{mm}}^{2}$) filtered with 504/12 excitation (Semrock FF01-504/12-25), FF518-Di01 dichroic (Semrock), and 532/18 emission (Semrock FF01-532/18-25). Images were acquired at 500 frames per second (fps) on a Kinetix sCMOS camera (Teledyne Photometrics) via Micro-Manager, with 2×2 or 4×4 binning, for 20 to 30 s per recording. All neuronal voltage recordings were acquired on a single z-plane at the recording plane.

### 4.10 Neuronal Voltage Imaging Analysis

Recordings were motion-corrected using rigid NoRMCorre within CaImAn^66^^–^^68^ and denoised with the SUPPORT pipeline^67^ (100 training iterations on a 5000-frame zebrafish tail dataset, 10 s). Regions of interest (ROIs) corresponding to individual cells were manually annotated and processed using the VolPy pipeline.^69^ VolPy parameters followed defaults except for a 1 Hz high-pass filter to optimize spike detection. Cells with well-defined spatial footprints after VolPy processing were retained for subsequent analysis. Spike detection threshold was set to SNR 3 (i.e., a spike was only labeled as a spike if, apart from being template matched in VolPy, it also had an SNR of 3 or higher) in every graphed trace. Only true detected spikes with SNR => 3 are used in all comparative and collective graphs in the manuscript, except in Fig. S4(e) in the Supplementary Material, where the spike detection threshold was set to SNR 2, to study the relation between spike signal-to-noise ratio and cell signal-to-background ratio in noisy environments. For Fig. 4 visualization, traces were high-pass filtered at 1 Hz for baseline correction and plotted as SNR (spike-peak amplitude divided by baseline standard deviation). Correlation matrices were computed as Pearson correlations of firing rates in 10 ms bins. For Fig. S4 in the Supplementary Material, signal-to-background ratio was computed as the average fluorescence of a cell divided by the average fluorescence of a donut-shaped mask around it, with a diameter two times bigger than the cell.

### 4.11 Voltage Imaging of Heart Dynamics

Embryos at 25, 48, and 102 hpf (separate individuals at each stage) were reared in egg water. The 25 and 48 hpf embryos were manually dechorionated, and then all the embryos from 25, 48, and 102 hpf were incubated with 100  μM *para*-aminoblebbistatin (pAB) (MCE, HY-111474) for 4 h to inhibit heart contractions. To limit blue-light–induced loss of drug effectiveness, embryos were handled under reduced light, the heart was located using a different wavelength when feasible, and voltage recordings were acquired with low excitation intensity and short acquisition windows. Residual motion was occasionally observed and is a practical limitation of in vivo wide-field recordings. During early development, zebrafish can remain healthy even with inhibited heart contraction;^70^ a drop of pAB was added 5 min before recording. Embryos were mounted in 3% low-melting agarose (Sigma-Aldrich, A4018). Single-photon imaging was performed by Nikon A1RMP with a 25×/1.1 NA water objective (Nikon CFI75 Apo, 25XC W). Illumination was conducted by a white LED at 0.6 to $1.5\;\mathrm{mW}/mm^{2}$ (SOLA Light Engine, Lumencor), filtered with a 504/12 excitation (Semrock FF01-504/12-25), FF518-Di01 518 dichroic (Semrock), and 532/18 emission (Semrock FF01-532/18-25). Images were acquired at 100 fps on a Kinetix sCMOS camera (Teledyne Photometrics) by Micro-Manager, with 2×2 binning for 10 s per recording, on a single z-plane at the recording plane. For Fig. 5 traces, photobleaching was corrected with a 1.5 s rolling mean, followed by 30 ms rolling mean smoothing. For the traces present in Fig. 5, ΔF/F was calculated as the difference between the trace fluorescence and the average cell fluorescence, divided by the average cell fluorescence. To verify viability and demonstrate blue-light reversibility of pAB, hearts were illuminated at $10\;\mathrm{mW}/mm^{2}$ through a Nikon FITC cube (480/30 excitation, 505 dichroic, 535/45 emission) for 10 s at the end of each session to observe the resumption of contractions.^44^ Activity extraction used a unified pipeline (NoRMCorre motion correction,^66^ SUPPORT denoising,^67^ VolPy event detection^69^). For footprint visualizations, cardiac voltage-time maps were computed as the temporal maximum ΔF/F within a 200-ms window (see discussion and Fig. 5 caption for interpretation).

### 4.12 Anesthesia and Euthanasia Compliance

For zebrafish embryos >3  dpf, euthanasia was performed by overdose immersion in buffered tricaine (250 to 500  mg/L, pH 7.0 to 7.5) with exposure maintained for ≥30  min, and then they were rapidly chilled (2°C to 4°C) to ensure irreversibility, in accordance with the AVMA Guidelines for the Euthanasia of Animals (2020).^71^

## 5 Appendix B: Supplementary Videos

The following videos are included.

• Video 1. Quiescent heart during imaging at 4dpf.
• Video 2. Restoration of heart contractions by blue light exposure at 25 hpf.
• Video 3. Restoration of heart contractions by blue light exposure at 48 hpf.
• Video 4. Restoration of heart contractions by blue light exposure at 102 hpf.

## Supplementary Material

10.1117/1.NPh.13.S2.S23205.s01

10.1117/1.NPh.13.S2.S23205.s1

10.1117/1.NPh.13.S2.S23205.s2

10.1117/1.NPh.13.S2.S23205.s3

10.1117/1.NPh.13.S2.S23205.s4

## Data Availability

All data underlying manuscript figures have been uploaded to the 4TU Repository and are available at DOI 10.4121/b9c28138-c971-455e-b4de-c8f29b1daa96. Plasmids generated are available on Addgene under IDs collected in Table S2 in the Supplementary Material. Voltage imaging analysis was performed with publicly available SUPPORT and VolPy code.
