# Supplementary material for: Zebrafish Ace2N-mNeon expression toolkit for in vivo voltage imaging of neuronal synchrony and cardiac maturation
Source: Neurophotonics. 2026 May 9;13(Suppl 2):S23205. doi: 10.1117/1.NPh.13.S2.S23205 (PMC13157250; doi:10.1117/1.NPh.13.S2.S23205)
Supplement: Supplementary file 1 [file NPh_013_S23205_SD001.pdf]

Supplementary Information

A zebrafish Ace2N-mNeon expression toolkit enables in vivo voltage imaging of neuronal synchrony and cardiac maturation

ZhenZhen Wu[1], Rui Oliveira Silva[1, 2], Ruya Houssein[1], Fabiola Marques Trujillo[1], Jordan Gotti[1], Srividya Ganapathy[1], Zhenyu Gao[2], Daan Brinks[1, 3†]

[1]Department of Imaging Physics, Delft University of Technology, Delft, The Netherlands

[2]Department of Neuroscience, Erasmus University Medical Center, Rotterdam, The Netherlands

[3]Department of Molecular Genetics, Erasmus University Medical Center, Rotterdam, The Netherlands

Table S1. Primer sequences used in this study. Sequences are listed 5'→3'. Uppercase denotes engineered sequence (e.g., restriction site/linker/overlap); lowercase denotes the template-annealing region.

Primer name	Target / Notes	Sequence (5′⟶3′)
Ace2N-mNeon-fwd (cmlc2)	Insert (Ace2N-mNeon) for cmlc2 construct	gccggccctccaaatcagcagacttaac
Ace2N-mNeon-rev (cmlc2)	Insert (Ace2N-mNeon) for cmlc2 construct	atttggagGGCCGGCCCAACTTTTCTATAC
cmlc2-fwd	cmlc2 promoter	cagccatCCATGGTGGCGATTCTCC
cmlc2-rev	cmlc2 promoter	gcgcgccGTTCACTGTCTGCTTTGC
Ace2N-mNeon-fwd (unc45b)	Insert (Ace2N-mNeon) for punc503 / unc45b construct	atttggagGGCCGGCCCAACTTTTCTATAC
Ace2N-mNeon-rev (unc45b)	Insert (Ace2N-mNeon) for punc503 / unc45b construct	acgtcagccatGGTCCAGCCTGCTT
unc45b-fwd (punc503)	unc45b 503-bp promoter	aggtgtaaACCCAGCTTTCTTGTACAAAG
unc45b-rev (punc503)	unc45b 503-bp promoter	acgtcagccatGGTCCAGCCTGCTT
Ace2N-mNeon-fwd (acta2)	Insert (Ace2N-mNeon) for acta2 construct	gctggaccATGGCTGACGTGGAAACC
Ace2N-mNeon-rev (acta2)	Insert (Ace2N-mNeon) for acta2 construct	agctgggtTTACACCTCGTTCTCGTAG
acta2-fwd	acta2 promoter	aggtgtaaACCCAGCTTTCTTGTACAAAG
acta2-rev	acta2 promoter	cagccatGGTCCAGCCTGCTTTTTTG
Ace2N-mNeon-fwd (elavl3)	Insert (Ace2N-mNeon) for elavl3 construct	ctgcagataattaccggtATGGCTGACGTGGAAACC
Ace2N-mNeon-rev (elavl3)	Insert (Ace2N-mNeon) for elavl3 construct	ccaggatccaccggtTTACACCTCGTTCTCGTAG
elavl3-fwd	elavl3 promoter	ACCGGTGGATCCTGGCCG
elavl3-rev	elavl3 promoter	ACCGGTAATTATCTGCAGGTGG
Ace2N-mNeon-fwd (neuroD)	Insert (Ace2N-mNeon) for neuroD construct	gcgccaccATGGGGACCTGGATGCTG
Ace2N-mNeon-rev (neuroD)	Insert (Ace2N-mNeon) for neuroD construct	agctgggtTTACACCTCGTTCTCGTAGC
neuroD-fwd	neuroD promoter	aggtgtaaACCCAGCTTTCTTGTACAAAGTGG
neuroD-rev	neuroD promoter	tccccatGGTGGCGCAGCCTGCTTTTTTG

Abbreviations: *cmlc2*, cardiac myosin light chain 2; *unc45b*, unc-45b (503-bp promoter, punc503); *acta2*, alpha-smooth muscle actin; *elavl3*, ELAV-like protein 3; *neuroD*, neurogenic differentiation 1.

Table S2. Plasmids constructed in this study.

Addgene ID	Plasmid name	Promoter	Insert	Backbone / system	Notes
253616	pTol2pA2_ubi_Ace2N-mNeon	*ubi*	Ace2N-mNeon	Tol2pA2	Ubiquitous expression
253664	pTol2pA2_cmlc2_Ace2N-mNeon	*cmlc2*	Ace2N-mNeon	Tol2pA2	Cardiomyocytes
253619	pTol2pA2_punc503_Ace2N-mNeon	*unc45b (503-bp; punc503)*	Ace2N-mNeon	Tol2pA2	Skeletal muscle / adaxial
253618	pTol2pA2_acta2_Ace2N-mNeon	*acta2*	Ace2N-mNeon	Tol2pA2	Smooth muscle/pericytes
253615	pTol2pA2_elavl3_Ace2N-mNeon	*elavl3*	Ace2N-mNeon	Tol2pA2	Pan-neuronal (postmitotic)
653617	pTol2pA2_neuroD_Ace2N-mNeon	*neuroD*	Ace2N-mNeon	Tol2pA2	Neuronal subsets

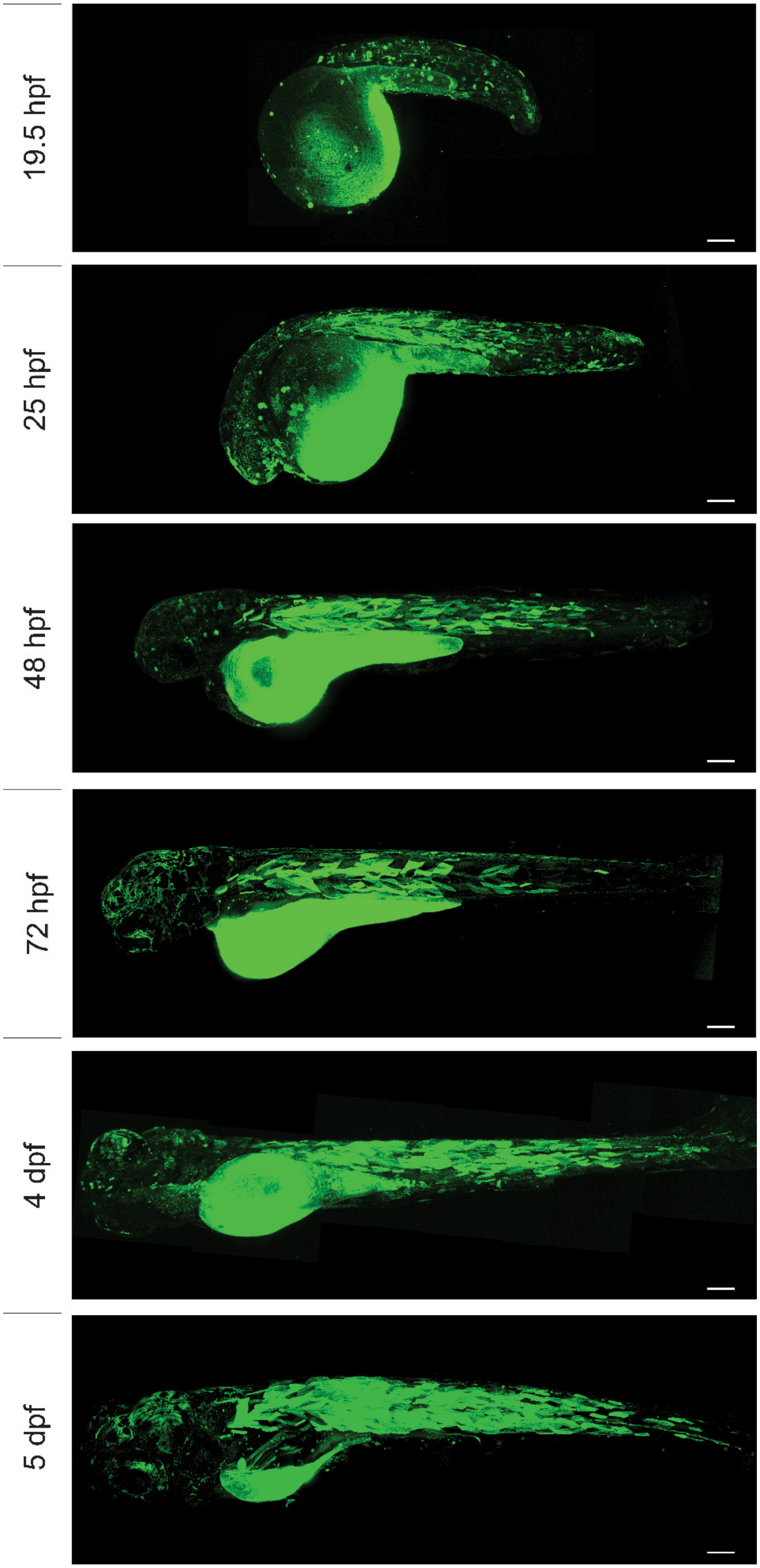

Figure S1. Higher-magnification views of Ace2N-mNeon expression driven by the ubiquitin promoter. *Single z-plane confocal images at the indicated developmental stages illustrate membrane-localized fluorescence across multiple embryonic tissues under the ubi promoter. Scale bar = 20 μm (unless indicated).* ***ubi:*** *ubiquitin;* ***hpf:*** *hours post-fertilization;* ***dpf:*** *days post-fertilization. Appended in native resolution at the end of the supplementary materials.*

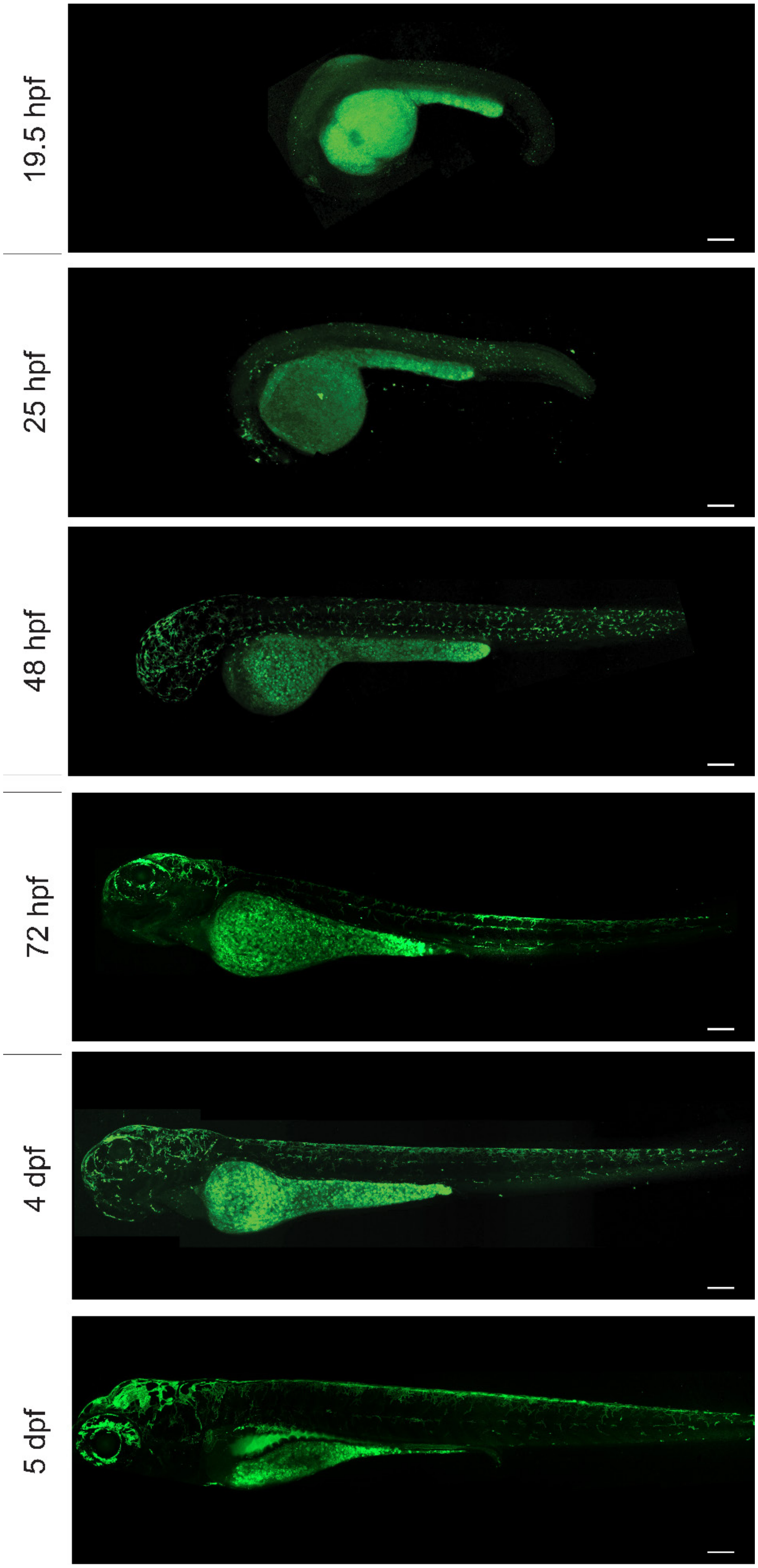

Figure S2. Tissue autofluorescence in uninjected embryos across development. *Representative fluorescence images from uninjected embryos during early development, showing baseline autofluorescence under the same imaging settings used for expression screening. Autofluorescence is visible in multiple regions, including the yolk-sac and parts of the head/brain.* ***hpf****: hours post-fertilization;* ***dpf****: days post-fertilization. Appended in native resolution at the end of the supplementary materials.*

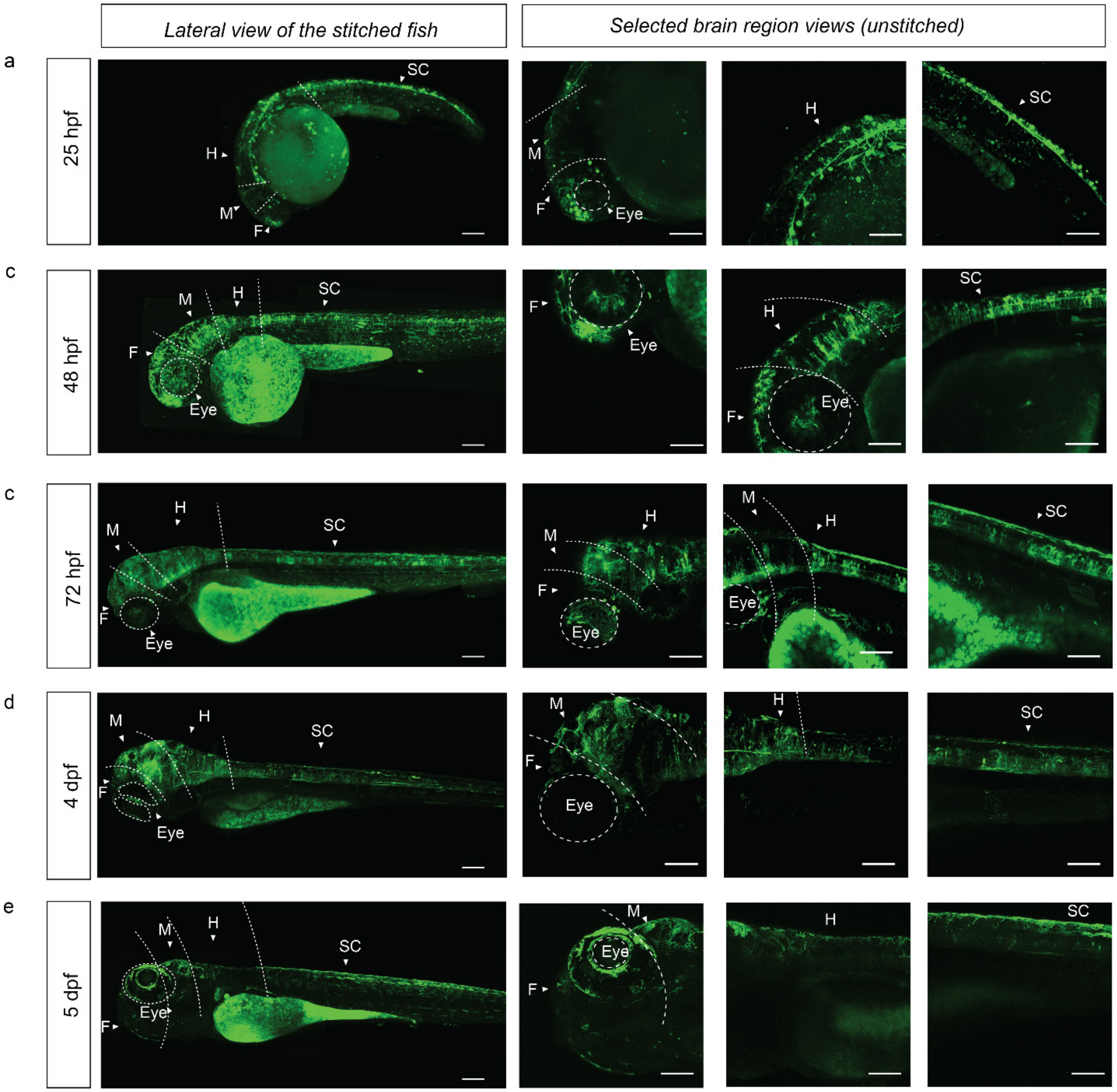

Fig. S3. elavl3-driven Ace2N-mNeon expression across brain regions during development. *Supplementary Fig. Sx | elavl3-driven Ace2N-mNeon expression across brain regions during development. Left, stitched maximum-intensity projections (lateral view) assembled from the confocal datasets shown in Fig. 3 to provide whole-embryo context and regional annotations. Right, unstitched regional views from the original acquisitions, shown at representative z-planes selected to best visualize membrane-localized signal in each brain region.* ***F****, forebrain;* ***M****, midbrain;* ***H****, hindbrain;* ***SC****, spinal cord. Brightness and contrast were adjusted for visualization. Scale bars, 100 µm for stitched projections and 50 µm for the regional views.*

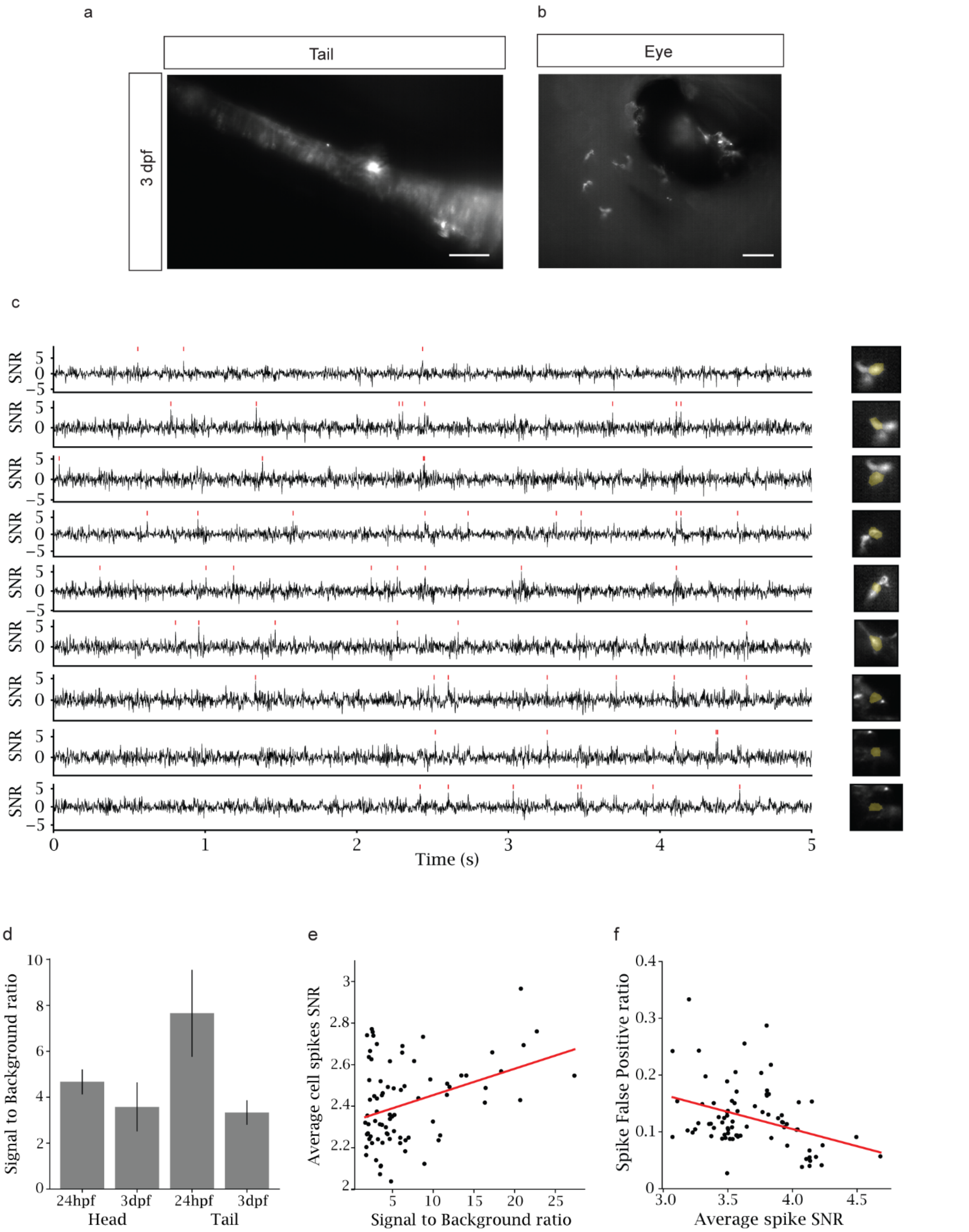

Figure S4. Voltage imaging in densely labeled neuronal fields. *(a–b) Representative FOVs at 3 dpf. (a) Tail: single-photon image showing high background and dense labeling, which limits suitability for voltage recording. (b) Head: single-photon image near the eye with neurons whose identity could not be confirmed based on morphology alone. (c) Example voltage*

traces extracted from several ROIs in panel b; red ticks indicate detected spikes. Right panels show the time-averaged image of the recording with manually annotated spatial footprints (shaded orange). Recordings were acquired as single z-planes; processing followed NoRMCorre–SUPPORT–VolPy as described in Methods. Scale bar = 50 µm. (d) quantification of changes in SBR (calculated as Fluorescence of the cells divided by the fluorescence of the surrounding Background) in head and tail between 1 and 3 dpf. (e) distribution of average spike SNR of individual cells as function of measured cellular SBR, with detection threshold of SNR 2, to showcase the correlation between spike SNR and cell SBR in noisy environments. (f) Distribution of the false positive spike detection ratio (i.e. ratio between spike detections in the inverted trace of a cell and spike detections in the native trace of the same cell) as function of the average spike SNR for the cell in which the spikes were detected, showcasing the negative correlation between false positive ratio and spike SNR. Data from panels (d) to (f) come from all cells in the recordings used for Figure 4 and Supplementary Figure 4. ***FOV:*** *field of view;* ***ROI:*** *region of interest;* ***dpf:*** *days post-fertilization;* ***SNR:*** *signal-to-noise ratio; Signal: peak fluorescence value of a spike in a trace; Noise: standard deviation of the resting fluorescence in a trace;* ***SBR:*** *Signal-to-Background ratio; Signal: Soma-averaged fluorescence (non-spiking); Background: fluorescence averaged over a donut shaped area surrounding the soma.*

Video S1-S3

Video. S1. Quiescent Heart during imaging at 4dpf

Video. S2. Restoration of Heart Contractions by Blue Light Exposure at 25 hpf.

Video. S3. Restoration of Heart Contractions by Blue Light Exposure at 48 hpf.

Video. S4. Restoration of Heart Contractions by Blue Light Exposure at 102 hpf.
